# Clinical features of delirious mania: a series of five cases and a brief literature review

**DOI:** 10.1186/1471-244X-12-65

**Published:** 2012-06-21

**Authors:** Bo-Shyan Lee, Si-Sheng Huang, Wen-Yu Hsu, Nan-Ying Chiu

**Affiliations:** 1 Department of Psychiatry, Lu-Tung Branch of Changhua Christian Hospital, Changhua, Taiwan; 2 Department of Psychiatry, Changhua Christian Hospital, Changhua, Taiwan; 3 Center of General Education, Central Taiwan University of Science and Technology, Taichung, Taiwan; 4 Chang Jung Christian University, Tainan, Taiwan

**Keywords:** Bipolar disorder, Delirium, Electroconvulsive therapy, Mania, Catatonia

## Abstract

**Background:**

Little is known about the cause and psychopathology of delirious mania, a type of disorder where delirium and mania occur at the same time. This condition still has no formal diagnostic classification. To provide more information about this potentially life-threatening condition, we studied five patients with delirious mania.

**Methods:**

We describe the cases of five patients with delirious mania admitted to an acute inpatient psychiatric unit between January 2005 and January 2007, and discuss the cases in the context of a selective review of the clinical literature describing the clinical features and treatment of delirious mania.

**Results:**

Two patients had two episodes of delirious mania. Delirium usually resolved faster than mania though not always the case. Delirious mania remitted within seven sessions of the electroconvulsive therapy (ECT).

**Conclusions:**

Delirious mania is a potentially life-threatening but under-recognized neuropsychiatric syndrome. Delirious mania that is ineffectively treated may induce a new-onset manic episode or worsen an ongoing manic episode, and the patient will need prolonged hospitalization. Delirious mania also has a close relationship with catatonia. Early recognition and aggressive treatment, especially with electroconvulsive therapy, can significantly reduce morbidity and mortality.

## Background

Concurrence of delirium and mania is unusual. While delirious mania is not described in the *Diagnostic and Statistical Manual of Mental Disorders*, fourth edition (*DSM-IV*), it does exist through the evidence of clinical reports. The syndrome was first described by Calmiel in 1832. In 1849, Luther Bell, who is credited with providing the first comprehensive description of the syndrome, reported 40 patients with the condition out of 1700 admissions to McLean Hospital [1]. Three-quarters of these patients died. In 1921, Kraepelin categorized mania into 3 types: acute, delusional, and delirious [2,3]. To better describe the range of severity, Klerman [4] described the spectrum of mania in 5 stages: normal, neurotic, hypomanic, manic, and delirious. However, the description of delirious mania provided in Klerman’s classification was very similar to the delusional type defined earlier by Kraepelin, and it would be coded as bipolar I disorder, manic episode with psychotic feature in the *DSM-IV.* Here delirious mania is defined much more closely to the extremely severe group in the final stage, stage III mania described by Carlson and Goodwin [5].

There is no clear consensus on the clinical characteristics associated with delirious mania or guidelines for treatment. Recognition of this syndrome is further complicated by the fact that many cases of delirious psychiatric patients are precipitated by medical or neurological conditions or use of psychoactive substances.

Other studies have shown a high incidence of disorientation, confusion, or delirium among manic inpatients, and have noted that these patients tend to be hospitalized longer than are manic patients without these additional symptoms [4-6]. These patients presented specific features other than a combination of typical mania and delirium. Karmacharya et al [1] mentioned some distinctive symptoms of delirious mania and described some severe forms of familiar manic/psychotic symptoms, which could provide clues about delirious mania.

Delirious mania is not recognized as a stand-alone diagnosis in the current nosology because many terms have been used over the years to describe patients presenting with mania, including excitement, delirium, and altered consciousness with/without catatonic symptoms. Other terms included lethal catatonia, malignant catatonia, delirious mania, and Bell’s mania [1,7]. Some authors have pointed to the high incidence of catatonic symptoms occurring in patients with delirious mania and also to the fact that both catatonia and delirious mania respond to electroconvulsive therapy (ECT) [8-10]. Taylor and Fink [10] provided a classification of catatonia, and suggested that it be reclassified as an individual abnormal behavior akin to delirium and dementia, while delirious mania was posited in the same subtype as delirious catatonia (delirious mania, excited catatonia) [10-12]. Karmacharya et al [1] did not find a large number of catatonic signs and symptoms in their study, while Detweiler et al [11] supported Fink’s proposal.

In an attempt to better describe the course of delirious mania and to provide a clearer picture of this illness, we present a series of 5 patients diagnosed with delirious mania. Our goal was to provide additional information about delirious mania and to illustrate the features of this condition and to highlight several challenges clinicians and researchers face in recognizing and treating this syndrome.

## Methods

We present the cases of five patients from our clinical practice at our ward. The criteria we used to select these cases include: (a) concurrent manic and delirious symptoms during hospitalization, and (b) medical workup failed to uncover an organic cause for either mania or delirium. Informed consent was obtained from the five patients for publication of this report.

## Results

Some specific features observed in these five cases were summarized in Table 1.

**Table 1:** Incidence of specific features observed

**Specific features**	**Incidence**
Having 2 or more episodes of delirious mania	2/5 (patient 1 and 2)
Delirium resolving faster than mania	4/5 (exception: 2^nd^ episode of patient 1)
Fulfilled Fink’s criteria of catatonia	2/5 (patient 2* and 3)
Fulfilled criteria of catatonia in DSM-IV	3/5 (patient 1*, 2* and 3)
Treatment of pharmacotherapy and ECT	2/5 (patient 3 and 4)
Delirious mania remitted within 7 sessions of ECT	2/2
Treatment: only pharmacotherapy	3/5 (patient 1, 2 and 5)
Delirious mania lasting over 2 months	1/3 (patient 1 had 2 episodes)

* = clinical symptoms on the borderline for specific symptoms of catatonia because of limited frequency and severity.

### Case reports

#### Patient 1

A 53-year-old male began acting oddly about 1 month prior to being admitted to the hospital. His family reported that his speech was pressured, and that he had begun wandering about without seeming to know where he was going. He also had outbursts of anger and exhibited destructive behavior as well. After burning newspapers at home and threatening his family, he was sent to the emergency room and was then admitted to the psychiatric ward.

His medical history indicated that he had been diagnosed with bipolar I disorder at age 22 and had been hospitalized several times for treatment of manic episodes. He had been followed up at our clinic since he was 37 years of age. At age 49, he presented manic symptoms, including hyperactivity, incomprehensible speech, talking to himself, uncontrollable emotional outbursts, and destructive acts. After admission, he continued to have disorganized speech patterns, distractibility, and agitation. He exhibited grandiosity, had auditory and visual hallucinations, and showed occasional catatonia-like signs of excessive motor movement, purposeless walking around with mundane postures, and inexplicably squatting at corners. Because of his severe and worsening disorientation and marked inattention, a diagnosis of delirium and bipolar mania was entertained. Results of a detailed physical examination were unremarkable. No fever was detected and his blood pressure was normal. Vitamin B12 and folic acid levels were within normal ranges, and his electroencephalograms were normal. As a result, the diagnosis was changed to delirious mania. The physician proposed ECT, but the patient’s family refused it.

The patient was placed on a regimen of haloperidol decanoate, 50 mg/wk; valproate, 1500 mg/day (level: 53.89 mEq/L to 95.35 mEq/L); lithium carbonate, 600 mg/day (level: 0.35-0.74 mEq/L); trihexyphenidyl, 10 mg, and chlorprothexine, 100 mg/day. In addition, flunitrazepam, 2-4 mg/day, was given for severe insomnia throughout the patient’s hospitalization, with episodic augmentation of dormicum, 15 mg/day, and trazodone, 100 mg/day. Clonazepam, 4-10 mg/day, was prescribed for agitation. We considered decreasing the dosage of the antipsychotics, to avoid neurotoxicity, but this became impossible because the patient became ever more agitated and had to be restrained for longer periods.

About 15 days after he was admitted, we noticed he had general weakness, and replaced clonazepam with lorazepam, 1-7 mg/day, according to the severity of agitation. During this period, 2-4 mg of lorazepam was injected intramuscularly nine times for controlling his aggressive agitation from day 2 to day 33. After day 33, chlorpromazine, 25-50 mg, was injected intramuscularly three times for aggressive agitation because lorazepam had produced only a limited effect. Trihexyphenidyl, 10 mg/day, was used before admission and continued because of concern about the increased risk of the extrapyramidal syndrome (EPS). The patient had no obvious symptoms of EPS. Constipation was observed episodically, so 24 mg/day of sennoside was given initially and then replaced by dulcolax, 10 mg/day. On the 71^st^ day after admission, the patient was discharged without full remission. This occurred against his clinicians’ advice, but his family thought he had been hospitalized too long and they felt he seemed less agitated. His discharge medications remained the same, with the exception of the addition of oral haloperidol, 15 mg/day. His symptoms remitted about 2 weeks after discharge. His family claimed he took his medication as ordered after discharge.

At the latest admission, the patient’s manic behavior warranted close monitoring and frequent seclusion. His speech was rambling and pressured. His mood was elevated, and he was distracted, hostile. He impulsively and inappropriately touched other patients but not in a sexual manner and had urinary and fecal incontinence. He occasionally walked around aimlessly and once more squatted at corners purposelessly, which seemed to be catatonia-like signs. His delirium was worsening, with disorientation as to time and place. All this made his medical care very difficult.

The results of laboratory studies, including thyroid function tests, were normal. Physical examination showed no signs of inflammation or infection. The patient’s vital signs were normal. The clinical impression was delirious mania. The family once more refused ECT.

In an attempt to control his behavior, valproate (level: 66.44 mEq/L to 105.29 mEq/L), and lithium carbonate (level: 0.81 mEq/L to 1.16 mEq/L), were used concurrently with augmentation of his antipsychotic medication. The dose was titrated to maintain the valproate level at around 100 mEq/L and lithium at 1 mEq/L for expected maximal therapeutic effect. Trazodone, 100 mg/day, and lorazepam, 4 mg/day, were administered for their hypnotic effect. In addition, the antipsychotics were immediately started after the patient was readmitted. The initial agent selected was olanzapine (15 mg for 5 days), and this was then changed to quetiapine (50 mg for 3 days) and zotepine (100 mg to 200 mg for 1 month) in order to make him slightly sleepy. The manic symptoms gradually improved: Young Mania Rating Scale (YMRS) score declined from 42 (on day 9), peaked at 46 (day 16), then fell to 20 (day 30), and remained between 15-24. The patient’s YMRS score was 15 at discharge, but his delirium did not resolve. He seemed sleepy most of the time, but was episodically agitated and uncooperative with his treatment. His inattention and agitation made oral intake difficult and led to frequent choking. Nasopharyngeal tube feeding was sometimes needed. The hypoactivity with his sleepy state and slightly rigid limbs made catatonia and EPS difficult to rule out. But he had neither other catatonic symptoms such as echolalia nor hypertension and fever. Concern about EPS, catatonia, and anticholinergic-induced delirium led us to change the zotepine to quetiapine (400 mg to 500 mg for the remaining month) and to reduce the trihexyphenidyl dosage, from the initial 10 mg/day to 2 mg/day for the remainder of his hospitalization. At the same time, lorazepam was injected intramuscularly for agitation control, at a dosage of 1 mg/day (given one time) and 2 mg/day, given seven times from day 2 to day 77.

For financial reasons, the family arranged to have the patient transferred to another hospital. At discharge, he still had occasional delirium but he was much more cooperative with staff members. Two months later, he returned to our clinic, and was in near-remission but he had no clear memory about his treatment at the other hospital and thus couldn’t provide any information about the regimen at the other hospital.

#### Patient 2

A 58-year-old male had a history of bipolar I disorder and was being treated at a local clinic. His treatment regimen included lithium carbonate, 1500 mg/day, carbamazepine, 600 mg/day; haloperidol, 2 mg to 5 mg/day (titrated as needed); and trihexyphenidyl, 5 mg/day. His performance at work had deteriorated for several months prior to admission. Six weeks prior to admission, he began to show emotional lability, talking to himself, laughing hysterically, and exhibiting unusual behavior, such as remaining naked for an inappropriately long time after bathing. He presented at an outpatient clinic, displaying mania and was in a generally disorganized condition. Both delirium and dementia were suspected. His medication was adjusted at the clinic after symptoms developed, so this ruled out drug-induced delirium. After receiving 150 mg of zuclopenthixol, he showed general weakness and disorientation at night. Four days prior to admission, he had been treated with risperidone, 4 mg, and carbamazepine, 200 mg, because it was felt that the EPS might have been induced by use of traditional antipsychotics. He was finally admitted to the hospital because of irritability, loose associations, and talking to himself.

The bipolar disorder could be traced back to age 29, when his manic behavior led to hospitalization for 1 month. During that episode, disorientation was also noted. His wife denied any knowledge that he had abused substances in the past.

On admission, the patient presented with mixed symptoms of delirium and mania. He was inattentive, disorientated as to time, place, and person, and unable to find his bed. He was awake all night, busy without clear goals, spoke incoherently and had decreased appetite and intake. He took boxing stances and pumped his thigh at times without reason, showing catatonia-like symptoms. Electroencephalographic (EEG) results showed poor alpha waves and mild cortical dysfunction. The neurologist thought this was a normal pattern frequently seen in aging patients and indicated no specific illness. A computed tomography (CT) scan indicated an “aging brain,” with mild ventricular dilatation and mild widening of the cortical sulci. Vitamin B12 and folic acid levels were within normal ranges. Thyroid-stimulating hormone (TSH) levels were also within normal limits, while free thyroxine (T4) levels were slightly lower than normal (0.69 ng/dL; normal range: 0.7-1.48 ng/dL). Delirious mania was considered the cause for his delirium because there was no acute precipitating physical illness. His family refused the suggestion of ECT because they were not familiar with it.

After this, the patient was treated with risperidone, 4 mg/day, and valproate, 500 mg to 1000 mg/day (level: 98.28 μg/mL). On about the sixth day of treatment, he became oriented. The manic picture became much clearer in the following days. On the 11th day, lithium, 300 mg to 900 mg/day, was titrated up because the patient had gone several consecutive nights without sleep. On the 15^th^ day, quetiapine, 600 mg/day, replaced risperidone (4 mg/day) for his insomnia. When daytime drowsiness occurred for 2 consecutive days, quetiapine was then replaced by olanzapine, 20 mg/day. For insomnia, estazolam, 4 mg, or flunitrazepam, 2 mg, was used, depending on the severity of the insomnia. But lorazepam, 2 mg/day, was given 3 times, and zolpidem, 10 mg/day, or zopiclone, 15 mg/day, was administered 17 times more episodically as the patient’s insomnia worsened. The patient was discharged in partial remission about 1 month later because the family chose to care for him at home. The regimen at discharge was olanzapine, 20 mg/day; flunitrazepam, 2 mg/day; trihexyphenidyl, 6 mg/day (successfully treating the drug-induced EPS and rigidity of limbs); magnesium oxide, 750 mg/day; and lithium, 900 mg/day. The patient’s mood stabilized over the next 2 months, and there was no recurrence of delirium.

#### Patient 3

A 67-year-old male began an active campaign for local elective office 1 month before being admitted to the hospital’s psychiatric unit. After 2 weeks of campaigning, he developed pressured speech and irritability, remained outside all day, and increased his normal intake of food and fluids. A week later he had insomnia, confusion, an unsteady gait, and frequent falls. On the day of admission, he was found kneeling in the street.

The patient had a history of peptic ulcer, pyloric stenosis, gallstones, and adhesive ileus that had been treated by vagotomy, gastrectomy, cholecystectomy, and enterolysis. His first episode of depression occurred at age 58. He was admitted twice for manic episodes, and the last psychiatric hospitalization was 5 years prior to this admission. His regular regimen included lithium carbonate, 600 mg/day; trihexyphenidyl, 2 mg/day; clonazepam, 3 mg/day; and diazepam, 4 mg/day. A month before admission, bupropion, 75 mg/day, and olanzapine, 10 mg/day, had been added to help stabilize his mood.

After admission, he had the typical picture of mania, with euphoria, pressured speech, and over-involvement with other individuals, and he created frequent interpersonal conflicts on the ward. Bupropion was discontinued immediately. Because of tongue dyskinesia, the olanzapine was changed to quetiapine, 400 mg/day. Three weeks later, delirium appeared. He became disoriented. He was frightened by visual hallucinations, and angry at the staff, whom he believed had transported him to “the train station.” His lithium level was 0.41 mEq/L to 1.18 mEq/L while he was hospitalized. His white blood cell count (WBC) was elevated to 18100/uL, Segment 83%, but there was no fever or evidence of infection. He had an inguinal hernia, which was believed to be the source of his leukocytosis, but the hernia could be moved in place by the surgeon without any sign of infection. Two weeks later, the WBC count decreased to 6900/uL without antibiotics. Thus, infection was ruled out as a possible cause for delirium. The results of electrolytes and renal and hepatic function tests were normal. Results of CT scans were unremarkable. On the 12^th^ day, an abnormal EEG was recorded, with mild-to-moderate cortical dysfunction without epileptic findings. The patient’s delirium persisted, along with persecutory delusions, fear of eating, and with agitation, kicking, and shouting. Delirious mania was suspected.

Bilateral ECT was begun on the 29^th^ hospital day, and performed 3 times a week. After the fourth session, the patient’s orientation improved, but there were also signs of depression. Before the patient presented with depressed mood, he accepted 2-mg injections of lorazepam for agitation. The injections were given 14 times from day 4 to day 34, and 2 doses were given before delirium occurred. After the fifth session of ECT, his depression worsened. He tried to leap from his bed in a suicide attempt after the sixth session of ECT. He was totally oriented after the seventh session, but his depression persisted. Paroxetine, 10 mg/day, was started after the 12^th^ ECT session, and lithium, 600 mg/day, was resumed after the 16^th^ session. The patient’s depression improved after 17 sessions of ECT, and resolved completely a month later. The discharge regimen included: alprazolam, 1.5 mg/day; paroxetine, 10 mg/day; lithium, 600 mg; zopiclone, 15 mg/day; and loperamide, 2 mg/day. Loperamide was added to the regimen because the patient had diarrhea when he ate watery foods without his dentures.

#### Patient 4

A 50-year-old female had a 15-year history of bipolar disorder, which had resulted in four previous hospitalizations for manic episodes. She was admitted to the nearby mental health institute after 1 week of recurrent mania. During that hospitalization, clinicians noted a change in her level of consciousness. She developed aspiration pneumonia with respiratory failure, and an endotracheal tube was inserted. The patient was uncooperative and removed the endotracheal tube. She was transferred to our medical department and seen in consultation by a psychiatrist.

Because of obvious manic symptoms of euphoria with auditory hallucinations, incoherent speech, disorganized behavior, and impulsively touching others’ faces, she was transferred to the psychiatric ward after 5 days of medical stabilization.

Her medication was changed from aripiprazole, 10 mg to 15 mg/day (titrated up to control manic symptoms), to quetiapine, 600 mg/day (replacing aripiprazole for insomnia); clonazepam, 3 mg/day; lithium, 600 mg/day (level: 0.2 to 0.47 mg); and lorazepam, 4 mg at bedtime. In addition, lorazepam, 2 mg/day, was intramuscularly injected on days 7 and 9 to control agitation. After this, the patient was calm for hours. In addition to her manic symptoms, she had prominent catatonic symptoms. She alternated between excitement and mutism. She also displayed episodic absent-mindedness, with a staring expression, echolalia, and echopraxia. Because of her refusal to eat or drink and frequent aggressiveness, she required nearly constant restraint of her hands and had to be fed via nasogastric tube. These catatonic symptoms progressed while delirious symptoms appeared as disorientation to time and place with chaotic consciousness 3 weeks after the onset of mania. Fever subsided before delirium appeared and the WBC count decreased from 13600 to 7100. A diagnosis of catatonia and delirious mania was made, and bilateral ECT was begun on the 10^th^ day of delirium. The delirium subsided after the first session of ECT and totally resolved after the second session, although mania and psychosis remitted after the sixth session.

#### Patient 5

A 59-year-old male was admitted to the hospital with elevated mood, irritability, irrelevant speech, hyperactivity, poor sleep, odd feelings he described as an “earthquake,” and auditory hallucinations.

He had a history of bipolar I disorder beginning at age 32. He borrowed money irrationally with unclear plans for its use, wandered around outside; slept poorly; and ate sugar canes at another person’s fruit farm without permission. His unusual symptoms occurred episodically, usually lasted about 10 days, and resulted in several hospitalizations. At age 52, he underwent a frontoparietal craniostomy because of an intracranial hemorrhage after a traffic accident. Severe recent memory impairment was observed after that accident. At age 54, he had a single transient ischemic attack with left upper limb weakness, but totally recovered. During outpatient psychiatric follow-up, he once overdosed in an apparent suicide attempt during a depressive episode. Because of weight gain and depression, his dosage of risperidone, 3 mg/day, was discontinued 10 months prior to admission. Aripiprazole, 20 mg/day, and paroxetine, 10 mg/day, were added to his treatment regimen, along with extended-release valproate, in a dosage of 500 ~ 750 mg/day, according to clinical response. However, drug compliance was poor throughout the course of bipolar disease in his life. The paroxetine was discontinued at a clinic visit 15 days before the patient was admitted after he had experienced 2 days of insomnia, irritability and grandiose thoughts.

When he was first admitted, aripiprazole, 20 mg/day, was changed to immediate-release quetiapine, 400 mg/day, to control insomnia while he was hospitalized. In addition, lorazepam, 4 mg, was administered for insomnia. Three days after admission, delirium occurred. He became disoriented about time and place. Inattention and irrelevant speech were apparent, but his orientation occasionally improved. A CT scan showed right temporoparietal region postcraniotomy and generalized brain volume loss with dilatation of sylvian fissures, cerebral sulci, and ventricles, consistent with damage following a past cerebrovascular accident (CVA). Results from biochemistry and hematologic examination were unremarkable. The patient’s symptoms improved without full remission. Against medical advice, his family insisted that he be discharged on the 10th hospital day. A week later, he was fully oriented but it took an additional month for his mood to return to baseline levels.

## Discussion

### Delirious mania in bipolar disorder

Because symptoms of delirious mania could be induced by other medical illnesses, the cases selected in this article were patients in whom the bipolar disorder (BD) was the primary cause of delirious mania.

Patient 1 was already ill and had not received medication when he was admitted to the hospital. Neuroleptic malignant syndrome could be ruled out from his two episodes because he had neither autonomic dysfunction nor lead-pipe rigidity of his limbs [9]. Thus, delirious mania was the most likely diagnosis for his delirious state. Within 4 years he had recurrent delirious mania. These two episodes produced very similar symptoms. Patient 2 also presented with mania with delirium, although there was a 30-year interval between the two episodes. Other cases of recurrent delirious mania have been reported [8,13]. These cases supported the theory that BD patients have a high risk of delirium because delirium could recur within the same patients.

Patients 3 and 4 had medical illnesses other than BD at admission. In such cases, it is difficult to make a differential diagnosis of the cause of delirium. The mild inguinal hernia in patient 3 and the resolution of pneumonia in patient 4 may have made them vulnerable to delirium [14]. However, these illnesses had been treated appropriately and presented no worsening signs that would have required more aggressive management. These patients’ obvious manic symptoms made delirious mania the most likely diagnosis. In patient 4, prominent catatonia was seen, and this was recognized as an important sign of delirious mania by Fink and Taylor [12]. Although patient 5 had a history of two episodes of stroke and a craniostomy, the absence of a new brain lesion on CT scans ruled out the possibility of post-stroke delirium. Patient 5 had poor drug compliance and he took a below-normal dose of medication. Thus, in his case delirious mania was unlikely to be caused by medication.

In the usual clinical practice, BD patients with other medical co-morbidities are not rare, like our patient 3, 4, and 5. Delirious mania can be easily misdiagnosed in some BD patients if they have chronic medical illness and present with acute-onset excited delirium, which could be difficult to differentiate from delirious mania. The poor drug adherence of bipolar patients would make even psychiatrists consider delirious mania as the very, very last possibility. It explained that there is a high incidence of delirium among BD patients (35.5%), but there was little in the literature relating to delirious mania [20].

### Delirious mania: A severe form of mania or another syndrome?

For more than 200 years, the acute onset of agitated dreamy or delirious states have perplexed clinicians. Called “onirisme” in the French literature, some authors still term this dreamy state oneirophrenia. The term “delirious mania” is used to describe manic patients who have delirious symptoms that occur and remit without other evident medical reasons [1,2,8,10,13,15-17]. Initially Kraepelin, and then Carlson and Goodwin [5] and Klerman [4] defined delirious mania as the most severe type of mania. In their concept, delirious mania is part of the spectrum of manic symptoms. Thus, its appearance was only a level of severity but not a different etiology. In 1980, Bond reported three cases of delirious mania, adding a more detailed clinical description of the disorder [13]. He noted that delirium and mania presented acutely and responded to the traditional treatment for bipolar mania. The definition of delirious mania still was limited to BD; however, many reports in the literature revealed similar symptoms in medically ill patients without a history of BD [18,19]. Taylor and Fink attributed delirious mania to a form of catatonia because of its good response to ECT and the high frequency of catatonic signs reported with it [8,10]. Thus, “delirious mania” has had a confusing history and meaning because like catatonia it was treatable with ECT, but it was coined from delirium and mania.

When only delirium subsided from delirious mania, we supposed that delirious mania was gone while mania remained. In patients 2, 4, and 5, delirious mania responded quickly to effective treatment. This pattern has been found in many case reports, no matter whether the treatment is ECT or just pharmacotherapy [8,13,15-17]. It took 2 more months for mania to remit in patient 2 after his delirium disappeared. But on the second admission of patient 1, when mania subsided, delirium persisted. This phenomenon may indicate delirious mania cannot be recognized as the most severe form of mania, as supposed by Carlson and Goodwin [5]. If we viewed delirious mania as a syndrome, as proposed by Fink and Taylor, it would be reasonable because the course of delirious mania and mania is not the same [12]. Taylor and Fink attributed delirious mania to a form of catatonia because of its good response to ECT and the fact that it was frequently accompanied by catatonic symptoms [8,9,11,12]. They assumed that delirious mania is prone to occur in patients with BD, but not solely in BD patients. Following this logic, delirious mania should not be recognized as a specific type of episode in BD. So delirious mania could be superimposed upon BD; in that case, all symptoms looked like the most severe form of mania, which Carlson and Goodwin had theorized [5]. In such cases, the effective treatment could quickly cure delirious mania but it took longer for mania to remit. In other cases, delirious mania could abruptly appear in stable BD patients or people without history of BD [13].

We noticed many literature reports in which patients remitted within few days under effective treatment, no matter what kind of treatment they received, leaving no residual symptoms [8,13]. We surmise that this is a sign of “simple” delirious mania, which occurs in BD patients without preceding mania and disappears without ongoing mania. Remitted patients may describe it as being like “a nightmare” [13]. But we supposed further that delirious mania itself might induce one manic episode or worsen the ongoing mania. And it is a rational explanation for why manic patients with delirium need longer hospitalization--besides the explanation that no effective treatment was given [20].

### Treatment

Many authors strongly emphasize the efficacy of ECT for treating delirium, no matter what the etiology is [8,9,14,16,21]. Two of our patients received ECT. In patient 4, delirium disappeared after two sessions of ECT, and her symptoms remitted after six sessions. In patient 3, after the fourth session of ECT, his orientation improved while mania shifted to depression. We know ECT can induce mania [22]. But no cases of ECT-induced depression have been reported. We theorized that delirious mania and the manic course subsided at the same time and mood coincidently switched to depression. Patient 3 required 17 sessions of ECT to become stabilized. The three other patients did not receive ECT treatment because of their relatives’ objections. In seven cases reported by Fink [8], Strömgren [16], Friedman et al [17], and Danivas et al [23], orientation cleared in five patients after two to three sessions of ECT, and symptoms stabilized in the other 2 patients after 6 sessions. Six patients in the Karmacharya study had dramatic improvement and some response within one to four sessions of ECT [1].

ECT is the treatment of choice for catatonia, regardless of the etiology. Catatonic signs are observed in a few cases of mania [23]. Fink [12] argued that it is of limited clinical value to differentiate malignant catatonia, excited catatonia, delirious mania, rapid cycling mania, and mania with psychotic features, since ECT is remarkably effective in relieving each of these syndromes [23,24]. One case of analgesic (ziconotide)-induced delirious mania was successfully treated with ECT [24]. In Karmacharya and colleagues’ review of cases [1], ECT was uniformly beneficial in cases of delirious mania.

High-dose benzodiazepines, especially one-time oral doses of lorazepam (3 mg to 4 mg) often lead to noticeable improvement, but not as reliably as does ECT [1]. In patients 1 and 2, at least one kind of benzodiazepine was given, at most three kinds, all during the course of treatment. From our clinical observation, our use of benzodiazepines didn’t lead to resolution of delirium and mania. But the dosage of benzodiazepines in these cases was lower than that suggested by Karmacharya.

Although traditional antipsychotics have been used broadly and successfully to treat delirium [25], some authors warn that with delirious mania, traditional antipsychotics should be avoided [1,7]. So it is inevitable to suspect that traditional antipsychotics delayed remission of delirious mania or even worsened it at the first admission for patient 1. At his second admission, use of antipsychotics was limited to new-generation agents, but delirious mania persisted just as in the first episode. New-generation antipsychotics with a mood stabilizer were used without ECT used in patients 2 and 5. Delirium ceased within 1 week of admission in patient 2 and after 2 more weeks in patient 5. The results in these two patients echoed those of some new reports that new-generation antipsychotics may be beneficial for delirious mania [1,26]. Our findings support Karmacharya and colleagues’ findings that antipsychotics and mood stabilizers alone are less effective in controlling the symptoms of delirious mania.

In patient 4, delirious mania was accompanied by prominent catatonic signs. This pointed out a dilemma: antipsychotics may be beneficial for delirious mania but they should not be used for catatonia [27]. In this case, the patient responded to ECT quickly under simultaneous use of antipsychotics. But in patient 1, though catatonic signs were not prominent as in patient 3, it is not known whether antipsychotics were not effective as in other cases, due to presence of catatonia. Though Karmacharya didn’t provide the catatonic signs of his patients, some features may be catatonia-like, for example, extreme psychomotor agitation (pacing, constant motion), and pouring water (on one’s own head or on the floor) [1]. Catatonia may be one possible cause for antipsychotics, especially typical antipsychotics, to be less effective or detrimental in treating delirious mania. This is a question that remains to be answered in future research.

### Delirious mania: Catatonia? delirium?

In the *DSM-IV*, catatonia is primarily categorized as a subtype of schizophrenia and as a specifier in mood disorders. In Fink’s proposed category, catatonia should be reclassified into a separate category that requires its own diagnostic criteria and treatment guidelines different from those for schizophrenia and mood disorders. Delirious mania should be recognized as a subtype of catatonia [10-12]. We used Fink’s criteria for catatonia to diagnose our patients (Table 1). We found that patient 2 and patient 3 fulfilled the diagnostic criteria. Using *DSM-IV* criteria, four of six episodes occurred among our patients. Catatonia was not very obvious in patient 1 and patient 2 (they were on the borderline of catatonia), while it was obvious in patient 4. We found that excessive motor activity was the most frequent catatonic sign in delirious mania. Both patient 1 and patient 2 had excessive motor activity while patient 1 had posturing at the first admission, and rigidity of limbs at the second admission. Patient 2 also had limb rigidity, although this was attributed to medication. Although our sample size was small and the review of case reports might be insufficient, the results were similar to those of Karmacharya et al. [1] We suggest a larger study to confirm this.

In Fink’s classification, the worst form of catatonia is malignant catatonia (other subtypes: neuroleptic malignant syndrome and serotonin syndrome). Delirious mania is posited in the milder group, including delirious catatonia and excited catatonia, rather than malignant catatonia. Another classification is the use of excited nonmalignant catatonia for delirious mania [28]. Here the term “delirious catatonia” seems very appropriate because it presents the possibility that delirious mania, which lacks the obvious catatonic signs, and excited catatonia, which lacks sufficient delirious signs (another term like catatonic mania), present two ends of the spectrum of delirious catatonia. In this category, they share the symptoms of excitement, which is similar to mania.

The above description raised the question of what is the line between catatonia and delirium?

In many reviews, delirium could be treated with ECT [17,24] and the most lethal form of catatonia, malignant catatonia, also meets the criteria of delirium [29]. Following this logic, although the definitions of delirium and catatonia had different criteria--delirium diagnosed mainly from consciousness aspect while catatonia from movement-- they may share something etiologically. Using this assumption, it makes sense that no matter whether the patient has mania with delirium, mania with catatonia, or both at the same time, the treatment is the same with ECT.

## Limitations

The five cases illustrate the challenges to be encountered in real-world clinical care of patients with delirious mania; however, the diagnostic workup procedures and criteria selecting and sequencing treatments were not uniform for each case. This may reflect the lack of universally accepted diagnostic criteria for delirious mania, and lack of widely adopted guidelines for its treatment. There is only anecdotal-level evidence upon which to base diagnostic and treatment recommendations. On this basis, detailed case reports and case series continue to be valuable for improving case detection in clinical practice and for providing preliminary guidance in managing delirious mania. However, more rigorous and systemic investigation is needed.

## Conclusion

Delirious mania is best viewed as a phenomenon different from the manic episode alone because it can persist when mania subsides and disappear when mania persists. Sometimes delirious mania may occur abruptly without an underlying manic episode, and it can be treated effectively and immediately without residual symptoms. Delirious mania also has a close relationship with catatonia. Because it can be effectively treated with ECT, physicians should be alert to this diagnosis.

## Abbreviations

BD: Bipolar disorder; CVA: Cerebrovascular accident; ECT: Electroconvulsive therapy; EPS: Extrapyrimidal syndrome; YMRS: Young Mania Rating Scale.

## Competing interests

The authors declare no competing interest.

## Authors' contribution

We declare that all the listed authors have participated actively in the study and all meet the requirements of the authorship. BSL designed the study and wrote the protocol, BSL, SSH, and NYC performed research/study, BSL managed the literature searches and analyses, BSL, SSH and NYC wrote the first draft of the manuscript. BSL and WYH did the revision of the manuscript. All authors read and approved the final manuscript.

## Pre-publication history

The pre-publication history for this paper can be accessed here:

http://www.biomedcentral.com/1471-244X/12/65/prepub
